# Supplementary material for: Soil microbiome analysis supports claims of ineffectiveness of Pseudomonas fluorescens D7 as a biocontrol agent of Bromus tectorum
Source: Microbiol Spectr. 2023 Dec 5;12(1):e01771-23. doi: 10.1128/spectrum.01771-23 (PMC10782950; doi:10.1128/spectrum.01771-23)
Supplement: Supplemental file — Detailed methods and results. [file spectrum.01771-23-s0001.docx]

**Supplementary Material**

**Title:** Soil microbiome analysis supports claims of ineffectiveness of *Pseudomonas fluorescens* D7 as a biocontrol agent of *Bromus tectorum*

**Authors:** Gordon F. Custer^a,b,c,d,e^, Brian A. Mealor^f,g,h^, Beth Fowers ^f,g,h^, and Linda T.A. van Diepen^a,b,h*^

**Authors’ affiliations:**

^a^ Department of Ecosystem Science and Management, University of Wyoming, Laramie, WY, USA

^b^ Program in Ecology, University of Wyoming, Laramie, WY, USA

^c^ Department of Plant Sciences, Pennsylvania State University, University Park, PA, USA

^d^  Huck Institutes of the Life Sciences, The Pennsylvania State University, University Park, PA, USA

^e^ The One Health Microbiome Center, Huck Institutes of the Life Sciences, The Pennsylvania State University, University Park, PA, USA

^f^  Department of Plant Sciences, University of Wyoming, Laramie, WY, USA

^g^ Sheridan Research and Extension Center, Sheridan, WY, USA

^h^ Institute for Managing Annual Grasses Invading Natural Ecosystems, Sheridan, WY, USA

*Corresponding author: LvD – Email: linda.vandiepen@uwyo.edu

**Keywords:** cheatgrass, downy brome**,** biological invasion, herbicide, invasive plants

Materials and Methods

To better understand whether the bioherbicide *Pseudomonas fluorescens* strain D7 can establish in soil under field conditions, a study was implemented in the fall of 2015 by the Bureau of Land Management and Johnson County Weed and Pest District (Buffalo, WY, USA). The site, outside of Buffalo, WY, USA, was invaded by the invasive annual grass *Bromus tectorum* (cheatgrass) and treated using a combination of *Pseudomonas fluorescens* strain D7, a bioherbicide approved for use on cheatgrass in rangeland systems, and the chemical herbicide imazapic. Bioherbicides are commonly applied with chemical herbicides (1). Pre-treatment vegetation observations place the site in a western wheatgrass (*Pascopyrum smithii*)/cheatgrass plant community. The dominant soil series on the site is a Cambria-Kishona loam that corresponds to a 10-14” Loamy Northern Plains ecological site (NRCS Soil Survey).

*Vegetation survey of the site*

Pretreatment vegetation cover was assessed using 15 locations randomly selected within the overall project site in November 2015. Plant foliar cover was estimated at each sampling location using three line-point intercept transects. At each interception point, the foliar canopy was classified as either cheatgrass, field brome (*Bromus arvensis*), or one of the following functional groups: perennial grass, perennial forb, annual forb, shrub, litter, or bare ground. These data were not used for statistical analysis or to compare before and after treatment as this was not the focus of our study and only serve to describe the vegetation at the site.

*Bioherbicide formulation and field applications*

*Pseudomonas fluorescens* strain D7 research inoculum was obtained from Verdesian Life Sciences (Cary, NC USA) in a freeze-dried formulation shipped under cold conditions at 4 °C. The formulated product contained 2 x 10^11^ *P. fluorescens* D7 cells per 0.1 g of product. Two treatment rates (e.g., 0.49 and 4.94 g/ha at 1x and 10x recommended field rates, respectively) were applied within a total water carrier volume of 112.25 L/ha of mixed solution. Treatment plots were larger than 100 m x 50 m and irregularly shaped due to the variation in the leading front of cheatgrass invasion. Applications were made with a pressurized ‘boomless’ herbicide spray nozzle mounted onto an all-terrain vehicle, delivering a 6.1 m swath width. This represents a standard method for herbicide application in rangeland areas. First, the chemical herbicide imazapic was applied at two different rates (52.6 and 87.7 g ai/ha) on November 24, 2015. Next, the 4.94 g/ha *P. fluorescens* D7 treatment was made on November 24, 2015. The 0.49 g/ha *P. fluorescens* D7 treatment was applied on December 5, 2015. Control plots received no *P. fluorescens* D7 or imazapic and were sprayed with only water at 112.25 L/ha. No positive controls were included in the experimental design (e.g., plating of inoculant to ensure viability).

*Soil collection and processing*

Soil samples were collected on May 31, 2017, approximately 18 months after treatment application. Within each treatment area (i.e., the two application rates of biopesticide x 2 application rates of chemical herbicide), five 1 m^2^ subplots were sampled using a small ethanol-sterilized shovel; an additional five 1 m^2^ subplots were sampled from the control treatment area. Within each subplot, three soil samples were collected to a depth of 10 cm and composited for a total of 25 independent samples. Samples were transported to the lab on ice and processed within 24 hours. Each composite soil sample was sieved using a 2 mm sieve, and a subsample of the sieved soil was stored at -80 °C for DNA extraction. All tools were cleaned between samples with 70% ethanol.

*DNA extraction and library prep*

DNA was extracted from 0.25 g of frozen sieved soil using the MO BIO PowerSoil (MO BIO, Carlsbad, CA) extraction kit according to the manufacturer’s protocols. DNA extracts were frozen at -20 °C until a polymerase chain reaction (PCR) could be performed on all samples. Bacterial diversity was assessed using the V4 region of the 16S rRNA gene of the bacterial genomes. This region was amplified using the 515F (5`-GTGYCAGCMGCCGCGGTAA-3`) (2) and 806R (5`-GGACTACNVGGGTWTCTAAT-3`) (3) primer pair. PCR conditions used for amplification were as follows: 98 °C for 30 sec, 30 cycles of 98 °C for 10 sec, 65 °C for 10 sec, 72 °C for 8 sec, and 72 °C for 5 min. PCR reactions contained 20 μL total volume and consisted of 0.2 μL Phusion high-fidelity DNA polymerase, 4 μL of 5x Phusion Green HF buffer, 0.4 μL deoxynucleoside triphosphates (10 mM), 14.4 μL diethyl pyrocarbonate-water, 0.5 μL of each forward and reverse primer (10 μM), and 1 μL of template DNA. Each PCR was performed in triplicate to reduce PCR biases associated with any single reaction. A 1.5% agarose gel was used to verify successful amplification in each reaction. Positive and negative controls were included during each PCR and were checked alongside samples on the agarose gels. PCR products of successful triplicate amplifications were combined and then cleaned using Axygen’s AxyPrep Magbead PCR clean-up kit according to the manufacturer’s instructions (Axygen Biosciences, Union City, CA). To prepare an equimolar mix of cleaned PCR products, DNA concentrations were checked using a dsDNA HS assay kit on a Qubit 3.0 fluorometer (Invitrogen/ Life Technologies, Carlsbad, CA). Equimolar amounts of DNA for each sample were combined into a single tube. Final libraries were submitted to the University of Minnesota Genomics Center and sequenced on an Illumina MiSeq platform with V3 chemistry producing 2 x 300 bp paired-end reads. No positive or negative controls were included in sequencing.

*Sequence data processing*

Raw sequence data were processed in R V4.1 (4). First, primers and adapters were removed using Cutadapt (v.3.7) (5). Next, in dada2 (6), trimmed reads were filtered using the filterAndTrim() function with the following parameters: truncQ = 2, truncLen = c(220,200), maxN = 0, and maxEE = c(1,2). Next, the frequency of sequencing errors was assessed using the learnErrors function with 1x10^8 bp. Sequences were then dereplicated, and sequencing errors were corrected using the derepFastq () and dada() functions. Paired sequences were merged with a minimum overlap of 12 bp, and no mismatches were allowed. Chimeras were removed using the “consensus” method, and non-chimeric sequences were then trimmed to only include sequences with a length within the expected range of 252-255 bp. Taxonomy was assigned using the Silva database (v. 138) (7).

Members of the family Pseudomonadaceae assigned by the Silva database were manually checked using BLASTN using both the refseq_rna and nt (nucleotide collection) database, with and without the organism filter set to Pseudomonas fluorescens, and the top 5000 results were screened for the species assignment “*Pseudomonas fluorescens*” at sequence similarity of 99% or higher.

*Statistical analysis of microbiome data*

Processed reads were tabulated and imported into Phyloseq (8) for statistical analyses. Any sequence read not assigned to bacteria at the kingdom level was removed before rarefaction. Samples were rarefied to 14,500 reads. The rarefied datasets consisted of 24 samples with one sample failing to sequence.

Samples were then grouped based on the rate of biopesticide application as this allowed us to address the main objectives of our study. All statistical analyses were carried out in R V4.1 (4). Significant differences in alpha (α) diversity metrics (Shannon diversity (H`) and richness) across the rates of *P. fluorescens* D7 were assessed using ANOVA (α=0.05). If the global test indicated a significant difference, post-hoc differences were determined using Tukey’s HSD (α=0.05). Differences in the relative abundances of amplicon sequence variants (ASVs) assigned to *Pseudomonas* among treatments were assessed using Kruskal-Wallis testing as the assumptions of ANOVA were not met. Bray-Curtis pairwise dissimilarities of community composition were visualized using NMDS, and significant differences among application rates (β-diversity) were determined using PERMANOVA and the multivariate extension of Levene’s test of multivariate dispersion (adonis and betadisper, respectively) (9).

Scripts used for sequence data processing and statistical analyses can be found at github.com/gcuster1991/pseudo_cheatgrass.git.

**Results**

*Raw sequence data processing*

Quality filtering and bioinformatic processing of sequence data retained 48.5% (519,819) of the total raw sequence reads (1,070,207). This resulted in an average final read depth of 20,793 (+/- 3256, SD) reads per sample. Rarefaction curves (Fig. S1.) indicate a plateau in observed ASVs at 5,000 reads and support rarefaction at 14,500 reads per sample.

*Beta Diversity*

Betadisper showed multivariate dispersion across the application rates to be insignificant (p > 0.05). PERMANOVA testing showed that the application rate of bio-pesticide had no effect on bacterial β-diversity (p = 0.19, F_2,22_ = 1.722) (Fig. S2), and the two application rates were no different from the controls.

*Alpha Diversity*

ANOVA and pairwise comparisons revealed a significant decrease (p < 0.05, F_2,22_ = 4.517) in the number of observed ASVs in the 4.94 g/ha *P. fluorescens* D7 treatment compared to the 0.49 g/ha treatment (Fig S3) (4.94 g/ha – 450 +/- 53, 0.49 g/ha – 526 +/- 69) (p < 0.05). However, there were no statistical differences in Shannon diversity (H`) among the two treatment groups and control plots (p = 0.15, F_2,22_ = 2.034).

*Screening of Pseudomonadaceae*

To search for *P. fluorescens* in our sequence dataset, all reads assigned to the family Pseudomonadaceae by the Silva database were selected and manually checked using BLASTN (Table S1). The top 5000 alignments from BLASTN for each Pseudomonadaceae ASV produced putative assignment to *P. fluorescens*for both ASV 1115 and ASV 2361. Additionally, we report no statistical difference in the relative abundance of these ASVs across the application rates (p > 0.05) (Table S2). The most abundant of these ASVs (ASV 1115) accounted for less than 0.01% of the total reads in our dataset, and the other two represented 0.009% and 0.005% (ASVs 1408 and 2361, respectively).

**Data availability**

Raw sequence data can be found under NCBI SRA project number PRJNA962488.

1. Tekiela DR. 2019. Effect of the Bioherbicide Pseudomonas fluorescens D7 on Downy Brome (Bromus tectorum). Rangel Ecol Manag https://doi.org/10.1016/j.rama.2019.10.007.

2. Parada AE, Needham DM, Fuhrman JA. 2016. Every base matters: Assessing small subunit rRNA primers for marine microbiomes with mock communities, time series and global field samples. Environ Microbiol 18:1403–1414.

3. Apprill A, Mcnally S, Parsons R, Weber L. 2015. Minor revision to V4 region SSU rRNA 806R gene primer greatly increases detection of SAR11 bacterioplankton. Aquatic Microbial Ecology 75:129–137.

4. R Development Core Team. 2020. A Language and Environment for Statistical Computing. R Foundation for Statistical Computing. Vienna, Austria.

5. Martin M. 2011. Cutadapt removes adapter sequences from high-throughput sequencing reads. EMBnet J 17:10.

6. Callahan BJ, McMurdie PJ, Rosen MJ, Han AW, Johnson AJA, Holmes SP. 2016. DADA2: High-resolution sample inference from Illumina amplicon data. Nat Methods 13:581–583.

7. Quast C, Pruesse E, Yilmaz P, Gerken J, Schweer T, Yarza P, Peplies J, Glöckner FO. 2012. The SILVA ribosomal RNA gene database project: improved data processing and web-based tools. Nucleic Acids Res 41:D590–D596.

8. McMurdie PJ, Holmes S. 2013. phyloseq: An R Package for Reproducible Interactive Analysis and Graphics of Microbiome Census Data. PLoS One 8:1–11.

9. Oksanen J, Blanchet FG, Friendly M, Kindt R, Legendre P, McGlinn D, Minchin PR, O’Hara R, Gavin L, Simpson P, Solymos M, Stevens HH, Szoecs E, Wagner H. 2017. vegan: Community Ecology Package. R package version 2.4-3.

*Table S1.* Sequences of Amplicon Sequence Variants (ASVs) assigned to the bacterial family Pseudomonadaceae by Silva taxonomic database. Sequences were then screened using BLASTN. A bold ASV ID indicates potential assignments to *P. fluorescens*.

| *ASV ID* | *Sequence* |
| --- | --- |
| **ASV 1115** | *TACAGAGGGTGCAAGCGTTAATCGGAATTACTGGGCGTAAAGCGCGCGTAGGTGGTTTGTTAAGTTGGATGTGAAATCCCCGGGCTCAACCTGGGAACTGCATTCAAAACTGACAAGCTAGAGTATGGTAGAGGGTGGTGGAATTTCCTGTGTAGCGGTGAAATGCGTAGATATAGGAAGGAACACCAGTGGCGAAGGCGACCACCTGGACTGATACTGACACTGAGGTGCGAAAGCGTGGGGAGCAAACAGG* |
| ASV 1408 | *TACAGAGGGTGCAAGCGTTAATCGGAATTACTGGGCGTAAAGCGCGCGTAGGTGGTTTGTTAAGTTGAATGTGAAATCCCCGGGCTCAACCTGGGAACTGCATCCAAAACTGGCAAGCTAGAGTAGGGCAGAGGGTGGTGGAATTTCCTGTGTAGCGGTGAAATGCGTAGATATAGGAAGGAACACCAGTGGCGAAGGCGACCACCTGGGCTCATACTGACACTGAGGTGCGAAAGCGTGGGGAGCAAACAGG* |
| **ASV 2361** | *TACAGAGGGTGCAAGCGTTAATCGGAATTACTGGGCGTAAAGCGCGCGTAGGTGGTTTGTTAAGTTGGATGTGAAATCCCCGGGCTCAACCTGGGAACTGCATCCAAAACTGGCAAGCTAGAGTATGGTAGAGGGTGGTGGAATTTCCTGTGTAGCGGTGAAATGCGTAGATATAGGAAGGAACACCAGTGGCGAAGGCGACCACCTGGACTGATACTGACACTGAGGTGCGAAAGCGTGGGGAGCAAACAGG* |

*Table S2.* Mean and standard deviation of rarefied read abundances by application rate for sequences of Amplicon Sequence Variants (ASVs) assigned to the bacterial family Pseudomonadaceae by Silva taxonomic database.

|  | ASV 1115 | ASV 1408 | ASV 2361 |
| --- | --- | --- | --- |
| *0.49 g/ha* | *0.0 (0.00)* | *1.9 (3.73)* | *0.0 (0.00)* |
| *4.94 g/ha* | *0.9 (2.85)* | *0.5 (1.58)* | *2 (4.32)* |
| *Control* | *6.2 (8.48)* | *2.2 (4.92)* | *0 (0.00)* |

*
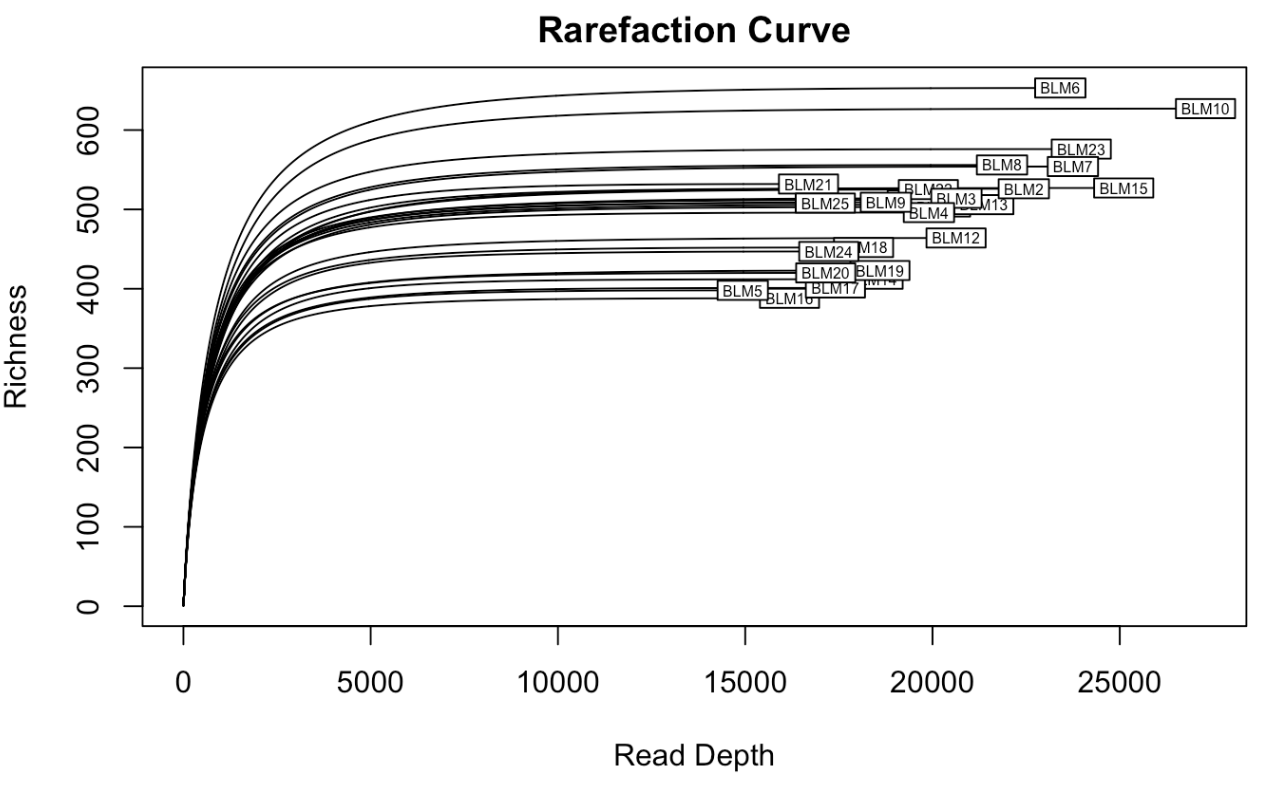
*

*Figure S1. Rarefaction curves show a plateau in observed ASVs at ~5,000 reads.*

*
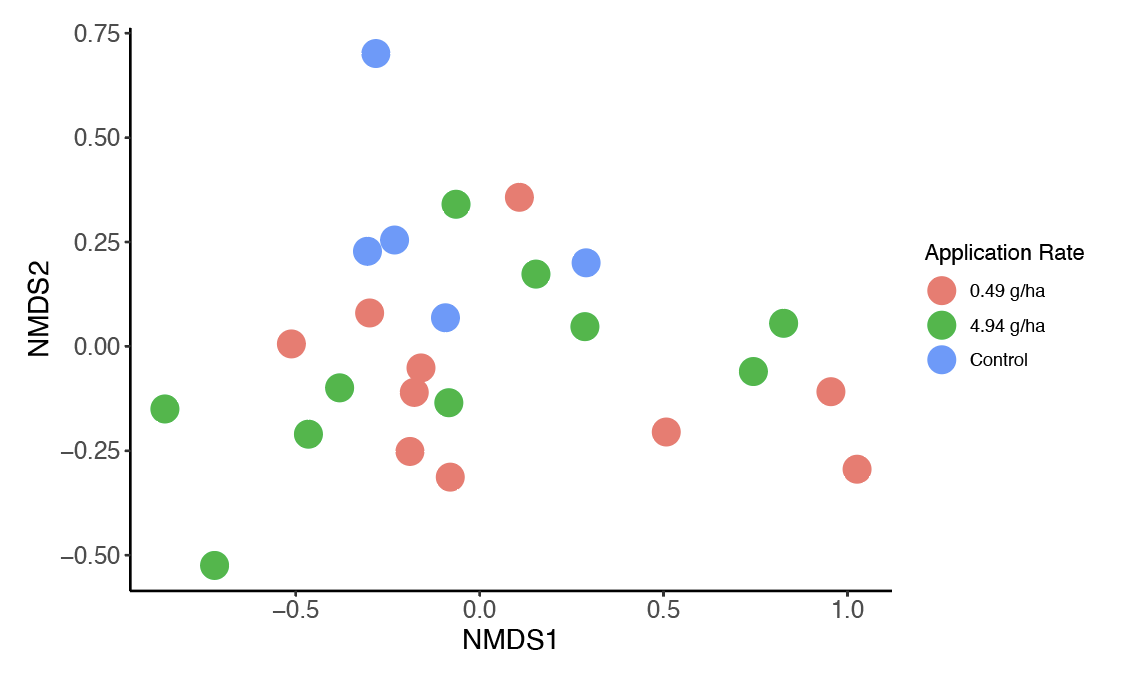
*

*Figure S2. Nonmetric multidimensional scaling (NMDS) of bacterial community based on Bray-Curtis dissimilarities. No significant differences among the treatment groups were found.*

*
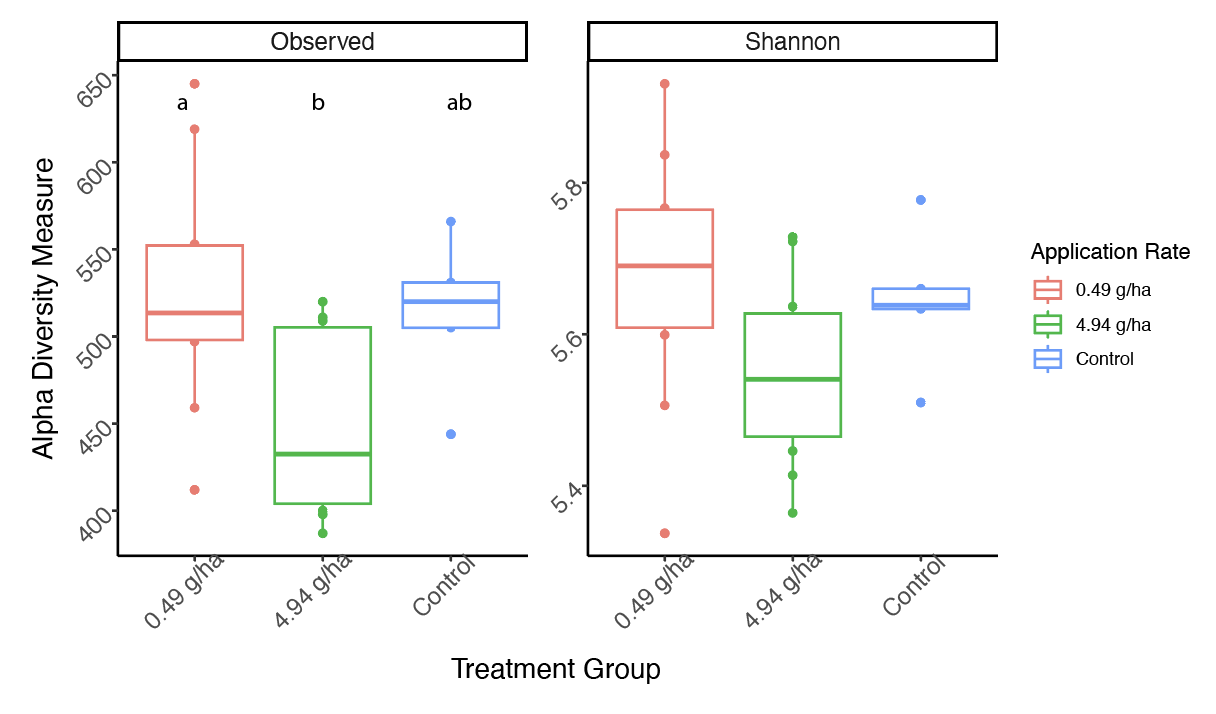
*

*Figure S3. Alpha diversity boxplots of treatment groups and control. A) Richness (observed number of ASVs), and B) Shannon diversity (H`). Letters indicate pairwise statistical significance at α = 0.05 as per Tukey’s HSD. Boxplots show the median (center line), 25^th^, and 75^th^ quartiles (top and bottom lines, respectively). Whiskers represent 1.5x the interquartile range (IQR).*
